# Accelerometer output and its association with energy expenditure in persons with mild-to-moderate Parkinson’s disease

**DOI:** 10.1371/journal.pone.0242136

**Published:** 2020-11-11

**Authors:** Brenda Jeng, Katie L. J. Cederberg, Byron Lai, Jeffer E. Sasaki, Marcas M. Bamman, Robert W. Motl

**Affiliations:** 1 Department of Physical Therapy, University of Alabama at Birmingham, Birmingham, Alabama, United States of America; 2 Graduate Program in Physical Education, Federal University of Triângulo Mineiro, Uberaba, Minas Gerais, Brazil; 3 University of Alabama at Birmingham Center for Exercise Medicine, University of Alabama at Birmingham, Birmingham, Alabama, United States of America; 4 Department of Cell, Developmental, and Integrative Biology, University of Alabama at Birmingham, Birmingham, Alabama, United States of America; 5 Department of Medicine, University of Alabama at Birmingham, Birmingham, Alabama, United States of America; 6 Department of Neurology, University of Alabama at Birmingham, Birmingham, Alabama, United States of America; 7 Geriatric Research, Education, and Clinical Center, Birmingham VA Medical Center, Birmingham, Alabama, United States of America; Linneaus University, SWEDEN

## Abstract

**Objective:**

This study examined the association between ActiGraph accelerometer output and energy expenditure across different speeds of walking in persons with Parkinson’s disease (PD), and further generated cut-points that represent a metric for quantifying time spent in moderate-to-vigorous physical activity (MVPA) among persons with PD.

**Methods:**

The sample included 30 persons with mild-to-moderate PD (Hoehn and Yahr stages 2–3) and 30 adults without PD matched by sex and age. All participants completed 5 minutes of quiet, seated rest and then underwent three, 6-minute bouts of walking on a treadmill at three different speeds relative to the individual’s self-selected pace. Activity counts were measured using an ActiGraph accelerometer worn at the waist level on the least affected side for persons with PD and the dominant side for controls. The rate of oxygen consumption, or energy expenditure, was measured using a portable, open-circuit spirometry system.

**Results:**

Our results indicated a strong association between activity counts and energy expenditure for persons with PD (*R*^*2*^ = 0.87) and controls (*R*^*2*^ = 0.89). However, the significant difference in slopes resulted in a lower cut-point of 1,354 counts·min^-1^ for persons with PD than the cut-point of 2,010 counts·min^-1^ for controls.

**Conclusion:**

Our results support the application of the disease-specific cut-point for quantifying the amount of time spent in MVPA using ActiGraph accelerometers among persons with mild-to-moderate PD. Such an application may provide accurate estimates of MVPA in this population, and better inform future research examining the possible determinants and consequences of physical activity as well as testing of interventions for changing MVPA in PD.

## Introduction

Parkinson’s disease (PD) occurs in approximately 1 million adults in the United States [1] and manifests in motor (tremor, rigidity, bradykinesia, postural instability, gait dysfunction) and non-motor (cognitive dysfunction, depression, sleep disturbances, psychosis) symptoms [2]. The motor control and gait dysfunctions, in particular, might influence the energetic cost of walking in PD [3]. These consequences of PD may further contribute toward loss of independence and reduced quality of life [2, 4].

There is substantial interest in physical activity as an approach for managing the consequences of PD [5, 6]. Physical activity is defined as any bodily movement produced by contraction of skeletal muscles that results in increased energy expenditure above resting values (one metabolic equivalent of task, or MET, is equivalent to one’s resting metabolic rate). There are numerous studies that have reported an association between physical activity and improvements in mobility disability, cognitive function, depressive symptoms, and quality of life in PD [7–10]. Nevertheless, there is considerable evidence that persons with PD participate in significantly less physical activity compared with adults of the general population [5]. This has prompted an interest in the development of interventions for promoting physical activity behavior in PD [6].

The study of physical activity in PD requires measures and metrics for quantifying the behavior that are calibrated against biological substrates such as oxygen consumption (VO_2_) as a measure of energy expenditure that corresponds with the intensity of movement. This has often been conducted based on the association between output from body-worn motion sensors such as accelerometers and VO_2_ using indirect calorimetry during bouts of physical activity across a range of physical stimuli differing in intensity of movement [11]. The association between accelerometer output and energy expenditure yields cut-point values for processing and classifying accelerometer output into the metric of moderate-to-vigorous physical activity (MVPA; ≥ 3METs) [12]. For example, one study enrolled 50 college-aged adults who undertook three, 6-minute bouts of slow walking, fast walking, and jogging on a motorized treadmill, and examined the association between activity counts per minute from the accelerometer data and VO_2_ from the last three minutes of each bout for steady-state metabolic data. The association resulted in the cut-point, or threshold, of 1,951 counts·min^-1^ for quantifying time spent in MVPA. The derivation of that cut-point has advanced our understanding of MVPA as a health behavior by prompting research examining the determinants and consequences of physical activity as well as interventions for increasing physical activity in the general population of adults in the United States [13, 14].

That same preparation for calibrating output from accelerometers has been applied in other populations such as persons with the neurological disease multiple sclerosis (MS) [15]; we note this research, as it has direct bearing for informing similar research in PD regarding a study design and approach for data analysis. For example, one study reported a differential pattern of association between accelerometer output and energy expenditure between persons with MS and controls, and this resulted in significantly different cut-point for MVPA in persons with MS and controls of 1,584 and 1,950 counts·min^-1^, respectively [16]. Importantly, the value for controls confirmed the cut-point from previous research [11], and this disease-specific cut-point has initiated a line of research examining determinants and consequences of MVPA in MS [15]. This cut-point has further informed the development of interventions that target increasing physical activity behavior in this population.

The study of physical activity in PD could benefit from similar research as done in the general population and persons with MS. Indeed, wearable technology such as accelerometers may have potential for better monitoring physical activity or other features specific to PD. However, there is a need for the development and validation of algorithms that accurately capture MVPA in this population [17], as PD symptoms (i.e., bradykinesia, tremors, gait dysfunction, postural instability) may result in different, possibly lower, cut-points that generated for adults from the general population. If studies utilize cut-points generated for the general population, rather than persons with PD, this may result in inaccurate measurements of physical activity in persons with PD. Of note, the relationship between physical activity and its correlates, such as symptom management and health outcomes, may be significantly underestimated if cut-points are not specific for PD.

To date, researchers have not calibrated the output of accelerometer data (i.e., counts per unit time) with an objective, biological variable that is a defining feature of physical activity and the intensity of movement, namely energy expenditure. This is essential for generating accurate accelerometer cut-points for quantifying the intensity of physical activity based on the metric of MVPA. We are aware of one study that developed a PD-specific ActiGraph accelerometer cut-point based on gait speed for persons with mild-to-moderate PD; however, the actual association between gait speed and energy expenditure for quantifying MVPA in PD is not clear, and the study and its methodology did not generate cut-points for MVPA based on the examination of the relationship between activity counts and energy expenditure [18]. This is important as energy expenditure is a gold standard measure of exercise intensity and necessary for generating cut-points for MVPA in PD based on accepted levels of the intensity of movement based on metabolic equivalents of task. The lack of disease-specific cut-points based on the relationship between activity counts and energy expenditure may further result in inappropriate conclusions and underestimate the accuracy of behavioral interventions in this population. Collectively, such an endeavor may facilitate the examination of physical activity, its disease-related determinants and consequences, and the development of behavior change interventions among persons with PD.

The present study adopted methods from previous research in person with mobility disability caused by neurological disease [19] and calibrated the output of accelerometers in PD by examining the association between activity counts and energy expenditure during bouts of treadmill walking in persons with PD and controls matched by age and sex. The study further generated cut-points based on the rate of activity counts per minute that can be applied for processing accelerometer data into time spent in MVPA among persons with PD compared with controls; the controls were necessary as a positive check on the experimental preparation in yielding similar cut-points as in the general population [11] and examining if the cut-points differ in PD. The generation of a PD-specific cut-point may result in a more accurate measure of time spent in MVPA in this population.

## Materials and methods

### Participants

Participants with PD were recruited through support groups, clinics, and community events in the Birmingham area. The inclusion criteria for persons with PD were diagnosis of PD, age of 50–74 years, Hoehn and Yahr stage 2 or 3, and ability to walk independently without the use of an assistive device; the restriction on age was necessary for avoiding aging influences on the outcomes of this study. Persons with PD were not excluded based on the presence of dyskinesia, but were excluded if they were not responsive to dopaminergic medications or had motor impairments due to neuroleptic medication or multiple strokes. Controls were recruited from community events and word-of-mouth. Control participants were matched on age (±5 years of a person with PD) and sex, and included based on age of 50–74 years, apparently healthy characterized by the absence of major cardiovascular, neuromuscular, and/or pulmonary disease, and ability to walk independently without assistive devices. Persons with PD and controls were excluded based on the presence of any medication (e.g., metformin) or diagnosis of a condition (e.g., diabetes) that significantly affects metabolism. The Physical Activity Readiness Questionnaire was included for screening and identified contraindications for exercise engagement [20]; persons who answered “yes” to two or more questions were asked to receive physician’s clearance prior to participation. The sample consisted of 30 persons with PD, and 30 controls were matched on age and sex; this is consistent with previous studies of MS [19] and other clinical populations (e.g., breast cancer survivors) [21].

### Protocol

The study protocol was approved by the University, Institutional Review Board, and participants provided written informed consent. Data were collected in the laboratory on a single visit. Participants first completed demographic and clinical information and self-reported level of physical activity based on the Physical Activity Rating (PA-R) questionnaire with scores ranging between 0 (Avoids walking or exercise) and 7 (Runs more than 10 miles per week or spends more than 3 hours per week in comparable physical activity) [22]. Height and weight were measured using a calibrated scale and stadiometer. Persons with PD underwent assessments of disease status, the Hoehn and Yahr rating and the Movement Disorder Society version of the Motor Examination from the Unified Parkinson’s Disease Rating Scale (MDS-UPDRS ME), performed by a certified member of the research team. Participants were fitted with the accelerometer and the portable metabolic system. All participants completed 5 minutes of quiet, seated rest for measuring resting energy expenditure, and then performed a 6-minute walking bout of over-ground and three, 6-minute walking bouts on a motor-driven treadmill (Trackmaster TMX428, Fullvision). The over-ground 6-minute walking bout was for determining comfortable walking speed, as this often corresponds with moderate intensity physical activity [23]. This was the basis for selecting the speeds for the three, 6-minute bouts of walking on the treadmill, as this permitted a manipulation of activity counts and energy expenditure based on previous research [19, 24] and known equations for the association between walking speed and energy expenditure [23]. Participants walked at 0.2 meters·sec^-1^ slower than comfortable walking speed, comfortable walking speed, and 0.2 meters·sec^-1^ faster than comfortable walking speed. The delivery of walking at three different speeds would presumably yield a linear increase of both activity counts and energy expenditure for persons with PD and the controls. There were 5 minutes of seated rest between each of the four, 6-minute walking bouts, and the order of treadmill speeds was not randomized or counter-balanced.

### Measures

#### Accelerometer

Activity counts were measured using the ActiGraph GT3X+ accelerometer, and accelerometers were calibrated by the manufacturer before the start of this study. This accelerometer is lightweight and small, and contains a solid-state digital accelerometer that generated an electrical signal proportional to the force acting on it along three axes. Based on previous research, we only included activity counts from the vertical axis [16, 18]; these counts directly align with vertical acceleration of the body during walking [25], and walking is the primary mode of physical activity in PD. Acceleration detection for the GT3X+ ranged in magnitude from 0.5–2.5g, and the frequency ranged from 0.25–2.50Hz, with motion outside of normal human movements rejected by a band-pass filter. The acceleration signal was digitized by a 12-bit analog-to-digital converter and was integrated over the preprogrammed epoch interval of 1s. The accelerometer data were downloaded via ActiLife software using a sample frequency of 100Hz and reintegrated into counts per 1-second epoch applying the low frequency extension, and then imported into Microsoft Excel for further processing. Activity counts were expressed as the average counts·min^-1^ across each walking bout. One accelerometer was placed along the anterior axillary line at the waist level on the least affected side for persons with PD and the dominant side for controls.

#### Energy expenditure

The rate of oxygen consumption (VO_2_), or energy expenditure, was measured using breath-by-breath analysis with a portable, open-circuit spirometry system (OXYCON Mobile, Vyaire). The metabolic system was calibrated prior to each participant’s visit based on the manufacturer’s recommendations. Participants wore a face mask and chest harness that held the metabolic system. We measured VO_2_ on 15-second intervals during the three bouts of treadmill walking. The outcome of interest was steady-state VO_2_, and this value was calculated by averaging the VO_2_ over the last three minutes of each 6-minute walking bout and expressed as mL·kg^-1^·min^-1^.

#### Data processing and analysis

The accelerometer and metabolic data per speed were imported into Microsoft Excel for processing per subject. We based the approach of data processing and analyses with previous research examining the relationship between accelerometer activity counts and energy expenditure in other clinical populations [21, 26]. We treated the data within subjects as dependent and data between subjects as independent to generate individual-level regression equations rather than creating a regression equation using all data from all subjects collectively; this approach avoids violating a key assumption of regression (i.e., independence between subjects because data of each subject would be stacked across speed). We estimated the squared multiple correlation coefficient (*R*^*2*^), intercept, slope, and cut-point for MVPA (i.e., ≥ 3METs based on American College of Sports Medicine) [23] from regression equations that established the linear relationship between activity counts and energy expenditure across three speeds of treadmill walking per individual. The individual-level cut-point for quantifying MVPA was estimated based on the substitution of 10.5 mL·kg^-1^·min^-1^ (i.e., 3METs) into the individual-level regression equation per participant using Microsoft Excel. The resulting parameters then served as data and averaged and compared for group-based values, in part, for the subsequent data analyses using SPSS ver. 25 (SPSS Inc.). The accelerometer and metabolic data from each treadmill walking bout were examined using two-way, mixed-factor analyses of variance (ANOVAs) with group (PD and control) as a between-subject factor and speed (slow, comfortable, fast) as a within-subject factor. The effect sizes for the ANOVAs were expressed as partial eta-squared (*η*_p_^2^) with values of .01, .06, and .14 interpreted as small, moderate, and large effects, respectively [27]. The *R*^*2*^, intercept, slope, and cut-point for MVPA computed using Excel per participant with PD or controls were analyzed using independent samples *t*-tests in SPSS.

## Results

### Descriptive statistics

The sample included 30 persons with PD and 30 age- and sex-matched controls. Descriptive statistics of participants in the two groups are provided in Table 1. The two groups were similar in age, sex, weight, height, and self-reported physical activity levels based on the PA-R. Persons with PD had a median(range) MDS-UPDRS ME score of 21(1–70), and this indicated mild motor impairment based on a comparison with the mean value of the general population with PD [28].

**Table 1:** Descriptive statistics of participants with Parkinson’s Disease (PD) and controls.

Variable	PD (n = 30)	Controls (n = 30)	*p*-value
Age (years)	64.4 (6.4)	63.5 (6.9)	0.59
Sex (M:F)	19:11	19:11	1.00
Weight (kg)	79.2 (16.3)	81.1 (15.3)	0.65
Height (cm)	170.6 (8.5)	174.2 (9.1)	0.12
PA-R	3 (0–7)	3 (0–7)	0.88
Disease Duration (years)	6.6 (5.2)		

*Note*: M = male; F = female; PA-R = Physical Activity Rating questionnaire. Values are presented as mean (SD) unless otherwise stated. PA-R scores are reported as median (range).

### Treadmill protocol

All participants completed the three trials of treadmill walking. The walking speeds for the overall sample and persons with PD and controls separately are provided in Table 2.

**Table 2:** Three walking speeds for the overall sample and persons with Parkinson’s Disease (PD) and controls.

Speed	Overall	PD	Controls
Slower	0.9 (0.2)	0.8 (0.2)	0.9 (0.2)
Comfortable	1.1 (0.2)	1.0 (0.2)	1.1 (0.2)
Faster	1.3 (0.2)	1.3 (0.2)	1.3 (0.2)

*Note*: Values are expressed as meters·sec^-1^.

### Accelerometer data

The accelerometer data are provided in Table 3. The ANOVA did not identify a significant interaction between group and speed on the rate of activity counts per minute (*F* = 1.15,*p* = 0.30,*η*_p_^2^ = 0.02). There was a main effect of speed on the rate of activity counts per minute (*F* = 439.42,*p*<0.01,*η*_p_^2^ = 0.88) and a nearly significant main effect of group (*F* = 4.02,*p* = 0.05,*η*_p_^2^ = 0.07). The main effect of speed indicated a linear increase in activity counts across speeds for both groups, and further indicated that the activity counts was lower in persons with PD than controls across all three speeds.

**Table 3:** Accelerometer (ActiGraph GT3X+) and metabolic (rate of oxygen consumption [VO_2_]) data for rest and three treadmill walking speeds in persons with Parkinson’s Disease (PD) and controls.

Group	Variable	Rest		Speed	
			Slower	Comfortable	Faster
PD (n = 30)	GT3X+	0 (0)	1,152 (845)	1,896 (1,073)	2,746 (1,263)
	VO_2_	3.5 (0.6)	11.5 (2.8)	13.1 (2.8)	15.6 (3.4)
Controls (n = 30)	GT3X+	0 (0)	1,409 (746)	2,216 (556)	3,174 (932)
	VO_2_	3.6 (0.9)	10.5 (2.1)	11.7 (2.0)	13.7 (2.5)

*Note*: Values are reported as mean (standard deviation). Accelerometer data are expressed as counts·min^-1^ and VO_2_ expressed as mL·kg^-1^·min^-1^.

### Metabolic data

The metabolic data are provided in Table 3. There was no difference between groups in resting metabolic rate (*t*(58) = –0.56,*p* = 0.58). The ANOVA identified a significant interaction between group and speed in energy expenditure (*F* = 4.21,*p* = 0.02,*η*_*p*_^2^ = 0.10); there was a greater difference in energy expenditure between PD and controls with increasing speed. There was a main effect of speed (*F* = 285.17,*p*<0.01,*η*_p_^2^ = 0.83) and group (*F* = 4.99,*p* = 0.03,*η*_p_^2^ = 0.08) on energy expenditure; this indicated that energy expenditure increased with increasing speed, and persons with PD had a higher energy expenditure than controls across all speeds.

### Association between accelerometer and metabolic data

**Fig 1:**
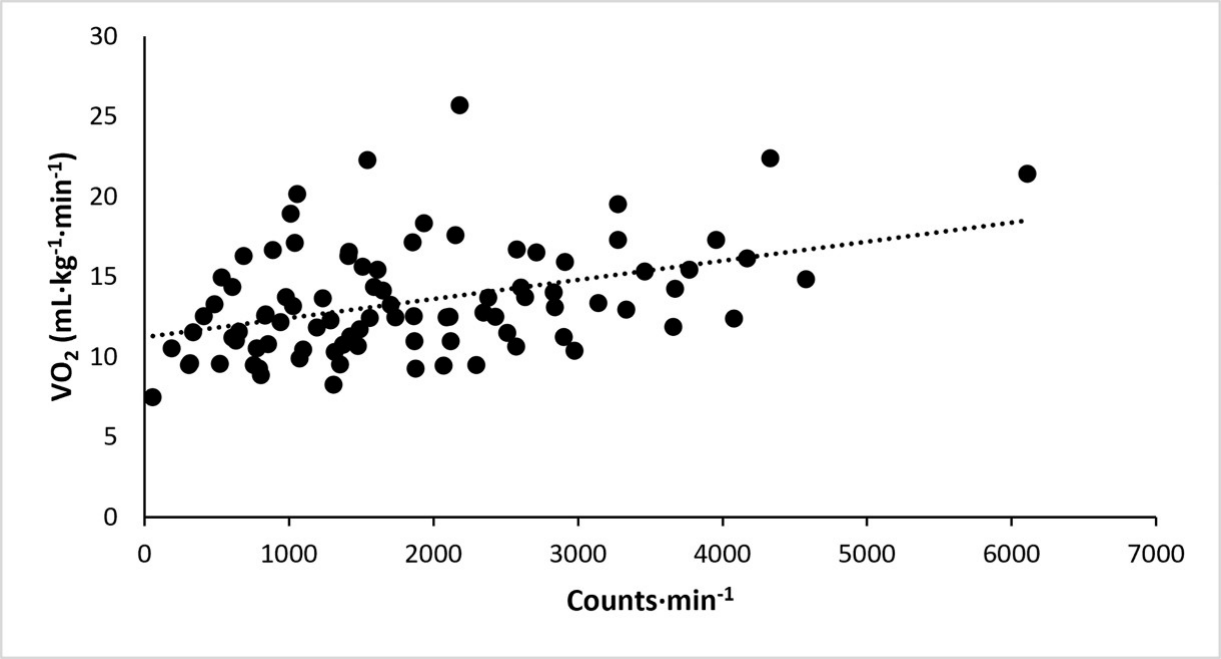
Scatter plot with a line of best fit for the relationship between activity counts and energy expenditure in 30 persons with PD.

There was a strong linear relationship between activity counts and energy expenditure in the overall sample based on the average *R* value(SD) of 0.94(0.12). The average *R*^*2*^ values for the association between activity counts and energy expenditure for persons with PD and controls were 0.87(0.12) and 0.89(0.12), respectively; these values were not different between the two groups (*t*(58) = 0.57,*p* = 0.57). The intercepts for persons with PD and controls were 4.998(1.375) and 4.382(0.910), respectively; those values were different between groups (*t*(58) = –2.05,*p*<0.05). The slopes between rates of activity counts and energy expenditure across speeds in persons with PD and controls were 0.005177(0.002858) and 0.003250(0.000894), respectively, and there was a steeper slope in persons with PD than in controls (*t*(58) = –3.52,*p*<0.01). We presented a scatter plot with a line of best fit for the relationship between activity counts and energy expenditure in persons with PD (Fig 1).

### Cut-points for moderate-to-vigorous physical activity

The mean(SD) cut-points for MVPA in persons with PD and the controls were 1,354(739) and 2,010(586) counts·min^-1^, respectively. There was a statistically significant difference between groups in cut-points for MVPA (*t*(58) = 3.81,*p*<0.01).

## Discussion

This study examined the association between accelerometer output and energy expenditure across three speeds of walking on a treadmill in persons with PD and age- and sex-matched controls. We further generated cut-points that represent a metric for quantifying time spent in MVPA among persons with PD. Our results indicated a strong association between activity counts and energy expenditure for both groups, but the slope of the association was steeper in persons with PD when compared with controls, which resulted in a lower cut-point in persons with PD. Overall, our results support the application of a disease-specific cut-point for better quantifying the amount of time spent in MVPA in persons with PD. Such an application may provide a more accurate estimate of physical activity in PD compared with controls. This will further better inform future research examining the determinants and consequences of physical activity and testing of interventions for changing MVPA in PD.

This is the first study to generate cut-points that represent time spent in MVPA based on activity counts and energy expenditure in PD. We identified a cut-point of 1,354 counts·min^-1^ for persons with PD, and this was significantly and substantially lower than the cut-point of 2,010 counts·min^-1^ for controls. To our knowledge, one study generated ActiGraph accelerometer cut-points based on gait speed from three, 3-minute bouts of self-selected brisk, normal, and slow over-ground walking and reported a cut-point of 1,316 counts·min^-1^ for persons with mild-to-moderate PD [18]; that value is comparable with the cut-point of 1,354 counts·min^-1^ in our sample with mild-to-moderate PD, as both studies recruited persons with comparable demographic and clinical characteristics who walked at similar speeds. However, the methodology of the aforementioned study included the use of Borg’s Rate of Perceived Exertion scale and heart rate, rather than indirect calorimetry for measuring actual energy expenditure, and further did not measure heart rate after the onset of steady-state energy kinetics [18]. Importantly, energy expenditure is a gold standard measure of the intensity of human movement and necessary for generating cut-points for MVPA in PD. Accordingly, we believe that our study provides novel and valuable cut-points for processing accelerometer data into time spent in MVPA based on the association with the biological substrate of energy expenditure.

Of note, we identified previous studies that have generated cut-points for persons with other neurological diseases such as MS. One study examined the association between activity counts and energy expenditure during treadmill walking and generated cut-points for quantifying time spent in MVPA (i.e., ≥ 3METS) among persons with MS who had minimal disability [16]. The cut-point for age- and sex-matched controls of the previous study was 1,950 counts·min^-1^ and was comparable with our cut-point of 2,010 counts·min^-1^; both cut-points for controls are consistent with previous research that reported 1,951 counts·min^-1^ for adults of the general population. Of note, the methodology of the aforementioned study and the present study involved treadmill walking using similar walking speeds. The pattern of results suggests that our methodology yielded observations that were consistent with previous research as a positive control. The cut-point for persons with neurological conditions followed a similar pattern such that persons with mild MS and mild-to-moderate PD had cut-points of 1,584 and 1,384 counts·min^-1^, respectively. Although both studies of MS and PD involved treadmill walking with comparable speeds, the cut-point for persons with PD was lower than persons with MS. This might be associated with the difference in age between the samples with MS and PD, as age is associated with lower cut-points in the general population of healthy adults [29].

There has been increasing interest in research on physical activity and its benefits for persons with PD, including the management of motor and non-motor symptoms, but the quality of this research has been undermined by problems with measurement of physical activity. For example, some researchers have utilized commercially available activity monitors, such as Fitbit and Garmin, that use proprietary algorithms for capturing physical activity levels not specific for the population with PD [30–32]. Of note, wrist-worn activity monitors may not necessarily capture physical activity levels given that persons with PD experience motor symptoms such as tremors and bradykinesia. If researchers utilize cut-points that are not specific to this population (i.e., those for healthy, older adults rather than PD), the levels of physical activity might be underestimated for persons with PD; this is problematic, as it might suggest a larger problem with inactivity in PD or underestimate the veracity of behavioral interventions in this population. Our study is first step in better understanding physical activity and its correlates and consequences by generating cut-points specific for persons with mild-to-moderate PD. This preliminary evidence can better inform the design of efficacious or effective interventions that examine physical activity as an outcome measure.

This study is not without limitations. One limitation is that our sample consisted of persons with PD who had mild-to-moderate motor impairment; this limits the generalizability of our results among the larger population of PD with more advanced motor impairment. Future research might consider generating cut-points for persons with PD who have higher disability status and use mobility devices such as bilateral walkers, as has been done in MS [19]. Of note, persons with PD came to the data collection session during the “on-medication” state for symptom management. One potential avenue of future research involves examination of how PD symptoms (i.e., bradykinesia, tremors, gait dysfunction) and fitness outcomes (i.e., muscular strength, postural control) might result in different cut-points in PD.

We believe that there are important avenues for future research applying this cut-point for persons with PD. For example, future research might provide a more accurate estimate of actual differences in levels of MVPA between persons with PD and controls or examine disease-related outcomes as possible correlates and consequences (i.e., disease progression, falls, and cognitive decline) of MVPA in PD. We further note that future research should consider examining theory-based variables as determinants of MVPA in PD, as there has been substantial interest in the application of theory-based behavioral interventions as an approach for increasing physical activity in persons with MS [33]. These examinations may lead to more well-informed and well-designed theory-based behavioral interventions and exercise training trials that target MVPA in persons with PD. Future research can use research-grade accelerometers to examine the accuracy and precision of commercially available activity monitors; this will allow researchers, clinicians, and exercise specialists to prescribe MVPA tailored for persons with PD.

## Conclusions

Overall, this study reports a strong linear relationship between activity counts and energy expenditure in the overall sample and subsamples of PD and controls. However, the cut-point for MVPA among persons with PD was lower than the value for controls, and the application of the cut-point may aid in more accurate measurements when quantifying time spent in MVPA in persons with PD. Such cut-points may further result in identifying determinants, tracking disease progression, and developing exercise interventions for the management of consequences in persons with PD.
